# Cost Analysis of Sentinel Lymph Node Biopsy for Oral Tongue Squamous Cell Carcinoma: Institutional Cohort and Population-Based Simulation

**DOI:** 10.1055/s-0045-1809906

**Published:** 2025-10-16

**Authors:** Hugo Fontan Köhler, Genival Barbosa de Carvalho, José Guilherme Vartanian, Luiz Paulo Kowalski

**Affiliations:** 1 Department of Head and Neck Surgery and Otolaryngology, AC Camargo Cancer Center, São Paulo, SP, Brazil; 2 Department of Surgery, Universidade de São Paulo, São Paulo, SP, Brazil

**Keywords:** mouth neoplasms, lymphatic metastasis, neck dissection, cost analysis

## Abstract

**Introduction:**

In oral carcinoma patients classified as cN0, selective neck dissection (SND) and sentinel lymph node biopsy (SLNB) may be used to stage the neck with equivalent oncological results.

**Objective:**

Compare the costs of SLNB and SND for oral squamous cell carcinoma.

**Methods:**

Analysis of institutional cohort and Markov chain model simulation using populational data.

**Results:**

We included 84 patients submitted to transoral resection and SLNB or SND and patients submitted only to SND. The mean cost was R$4,943,67 for SLNB and R$ 11,005.49 for SND with significant differences in length of stay (one versus three days, p < 0.001), operative time (92 versus 177 minutes, p < 0.001) and postoperative hospital visits in 60 days (two versus eight, p < 0.001). For the simulation model, the probability of not finding the SLN ranged from 0.0% to 5.7% with 0.5% increments and the probability of occult neck metastasis ranged from 9.0% to 100.0% with 1% increments. The costs of SLNB increase progressively as the rate of occult neck metastasis increases. When this rate reaches 56%, the cost difference becomes not significant. With rates above 72%, SLNB becomes significantly more expensive than SND. Using a public database, we calculate a cost decrease ranging from 27.93% to 66.54% with SLNB adoption.

**Conclusion:**

SLNB adoption may significantly decrease the costs associated with early-stage oral cancer treatment. It would allow more patients to be treated with the same number of resources now available.

## Introduction

According to the International Agency on Cancer Research (IARC) in its report on cancer incidence, 377.713 new cases of oral and lip carcinoma will be diagnosed in 2021 accounting for 177.757 deaths. 1 The Brazilian Cancer Institute (INCA) considers oral squamous cell carcinoma (OSCC) the sixth most frequent cancer in Brazilian males with a significant impact on mortality. 2 Multiple international guidelines suggest upfront surgery as the treatment of choice for patients with stage I and II. It consists of the removal of the primary tumor with adequate surgical margins and treatment of the neck lymph nodes by observation, selective neck dissection (SND), or sentinel lymph node biopsy (SLNB). 3

Neck treatment is directed by the estimated risk of occult lymph node metastasis with a 20% threshold conventionally accepted for elective surgery indication. 4 Recently, two prospective randomized clinical trials compared SND to observation and demonstrated a significant survival advantage of the former. 5 6 But SND also has significant morbidity with shoulder function impairment and shoulder and neck pain at arm exertion. 7 To minimize these risks, the concept of SLNB was applied to OSCC. 8

A European prospective cohort evaluated the diagnostic profile of SLNB when compared to SND and demonstrated an 86% sensitivity with a negative predictive value (NPV) of 95% with minimal morbidity and considered the procedure oncologically sound. 9 In an American multicentric prospective study, the NPV was 94% with routine pathological evaluation and increased to 96% with the addition of immunohistochemistry. 10 Two meta-analyses compared the NPV of SLNB and END in OSCC. Liu et al synthesized the results of 66 articles including 3,566 patients. The sentinel node was found in 96.3% of patients with an NPV of 94%. 11 The meta-analysis by Yang et al encompassed 35 articles with 1,084 patients with a successful SLNB in 98% of patients and NPV of 96%. 12 Finally, two prospective randomized trials compared SLNB versus SND regarding survival outcomes and quality of life (QoL). A French study demonstrated the equivalence of oncologic results in overall and recurrence-free survival. QoL outcomes were significantly better in the SLNB group after six months but equivalent at 12 months. 13 A Japanese study with a similar design had a similar result regarding oncologic outcomes but a sustained significant improvement in QoL after 12 months. 14 The financial costs of SLNB were compared to SND in two retrospective studies. Using the cost parameters and methodology of the Japanese health system, SLNB caused a significant cost reduction when compared to SND. 15 A Dutch study found similar results favoring SLNB. 16

Although SLNB is already in use in Brazil, 17 18 the costs associated with this treatment modality were not evaluated. Our objective is to evaluate the cost of SLNB in the Brazilian reality using data from a single institution to generate the monetary data and public information about the stage at diagnosis distribution of patients with OSCC to simulate an estimation of cost change with the adoption of SLNB.

## Methods

We analyzed patients treated at a single tertiary institution. Three patient cohorts were included: patients submitted to transoral OSCC resection and SLNB with prospective collection of clinical, pathological, and cost data; patients submitted to transoral OSCC resection and SND with prospective collection of clinical and pathological data and retrospective collection of cost data and patients submitted to metachronous SND after transoral resection with prospective collection of clinical and pathological data and retrospective collection of cost data. The purpose of this third cohort is to provide cost data on patients treated SLNB and afterward submitted to SND due to a metastatic lymph node.

Inclusion criteria were primary oral tongue tumors classified as cT1 and cT2 and no evidence of metastatic cervical lymph node after clinical and radiological evaluation by computer tomography or nuclear magnetic resonance. Patients with a history of previous malignant neoplasms in the head and neck area, previous treatment of the index tumor, or submitted to SND by alternative access routes were excluded from analysis. Patients were paired in the 1:3 proportion regarding neck treatment based on age, gender, comorbidities, and CT stage.

**Fig. 1:**
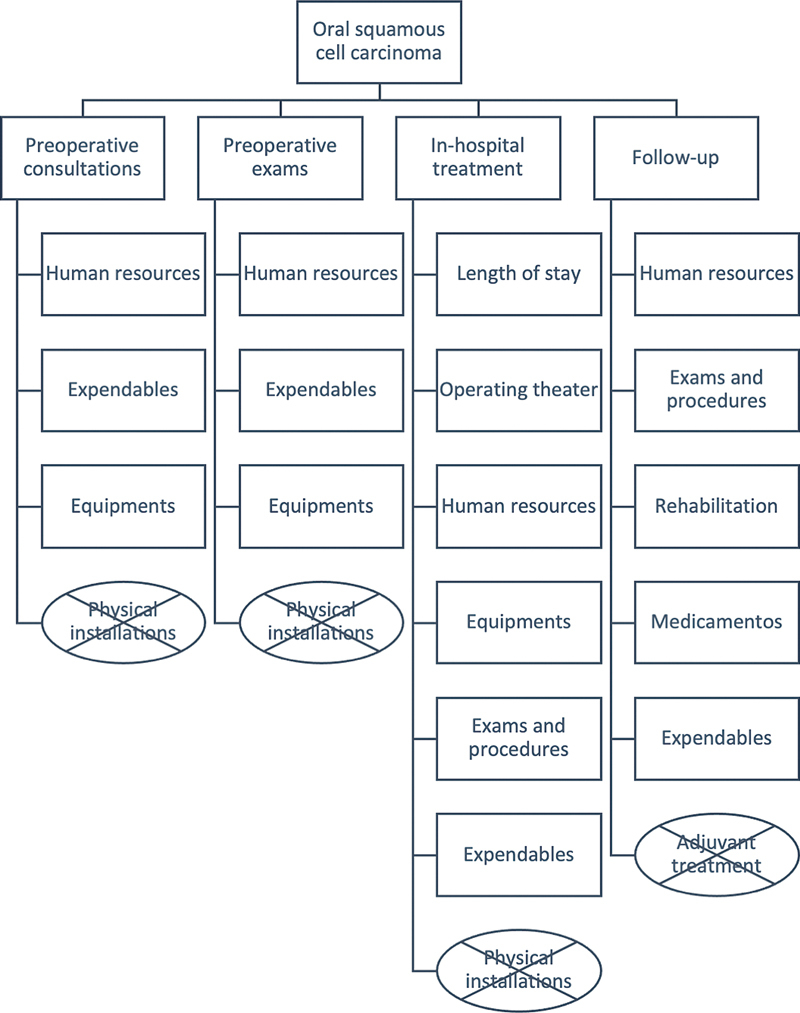
Direct and indirect costs of oral squamous cell carcinoma treatment. Costs not included in the analysis are specified in crossed rectangles.

We used a bottom-up cost analysis with the creation of itemized lists of material and personal resources used during treatment, including preoperative staging and preoperative exams, medical and paramedical costs, structural costs, and rehabilitation. The price attributed to each item was retrieved from the Electronic Auction Site (Bolsa Eletrônica de Compras do Estado de São Paulo, www.bec.sp.gov.br ). If the item was not found, the Health Ministerium price information was used. Hospital costs were extracted from the DataSUS (www2.datasus.gov.br). Since adjuvant treatment depends on the pathological data but not on the surgical procedure, we chose not to include it in this analysis. We used a time horizon of 60 days after surgery. All costs are expressed in Brazilian real (R$) and when the item price was not published in the current year, we used the General Price Index (Índice Geral de Preços, IPCA-E) for value correction since this index incorporates the health sector in its composition. Fig. 1 demonstrates the costs that were included or excluded in this analysis.

All data analysis was performed using the R software ( www.cran.org ). Continuous variables are described by median and range or mean and standard deviation (SD). Student t-test was used for mean comparison while the Mann-Whitney test was used for median comparison. A Markov model was designed to compare multiple possible situations. In this model, the probability of not finding the sentinel lymph node and the probability of a metastatic sentinel lymph node were independently varied to evaluate and compare the cost of each strategy. For each model change, 1,000 individual patients were simulated. Based on the prospective trials by Garrel et al 13 and Hasegawa et al, 14 oncologic results of both treatments were considered equivalent. All analyses were performed based on the intention to treat, therefore patients allocated to SLNB but submitted to SND were computed in the SLNB arm.

This study was submitted to the Institutional Review Board (Protocol Number 3633/24).

## Results

**Fig. 2:**
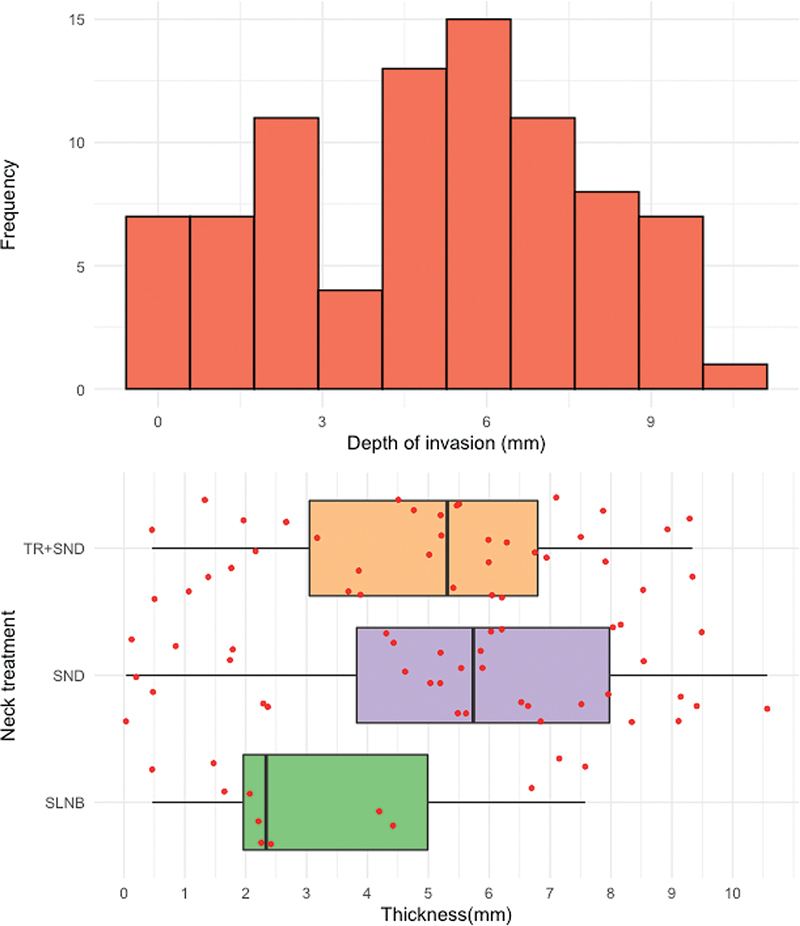
Histogram of depth of invasion in the entire cohort and boxplot for each treatment.

**Fig. 3:**
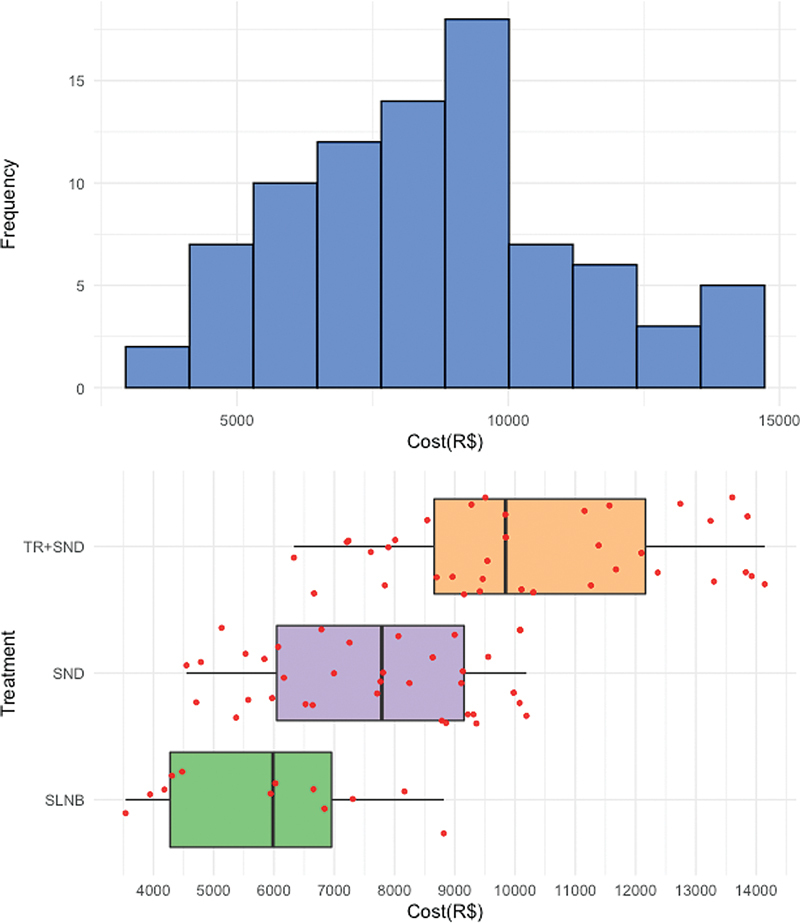
Histogram representing the cost distribution in the entire cohort and according to neck treatment.

We analyzed 84 patients in this study, 12 consecutive patients in the SLNB group, 36 consecutive patients in the simultaneous transoral resection SND group, and 36 patients in the delayed SND group. Demographic, clinical, and pathological data of patients included in this analysis are shown in Table 1 . Depth of invasion (DOI) distribution is shown in Fig. 2 for the entire cohort and each treatment group. Cost distribution according to treatment modality is shown in Fig. 3 . The mean cost in the SLNB group was R$ 4.943.67 and in the SND group was R$ 11,005.49 (p < 0.001). Treatment costs of the primary tumor are included in these values. Variables with a significant difference between groups were hospital length of stay (one versus three days, p < 0.001), surgical theatre use (92 minutes versus 177 minutes, p < 0.001), and number of hospital visits in 60 days (two versus eight, p < 0.001).

**Table 1:** Clinical and demographical characteristics of patients included in this cohort according to treatment modality

Variable	Values	SLNB	Transoral resection + SND	Isolated SND
Number of patients		12	36	36
Age	Mean/SD	53,2 / 10,5	54,1 / 6,7	54,2 / 8,0
Gender	Female	6 (50,0%)	20 (55,5%)	19 (52,8%)
	Male	6 (50,0%)	16 (44,5%)	17 (47,2%)
Number of retrieved nodes	Minimum/ Maximun/ Median	1 / 1 / 3	26 / 36 / 45	22 / 33 / 49
Cost (R$)	Mean/SD	5.782,49 / 1.767,17	11.117,25 / 2.033,14	7.620,43 / 1.750,17
cT classification	1	8 (66,7%)	19 (52,8%)	15 (41,7%)
	2	4 (33,3%)	17 (47,2%)	21 (58,3%)
cN classification	0	12 (100,0%)	36 (100,0%)	36 (100,0%)
pT classification	1	5 (41,7%)	20 (55,5%)	14 (38,9%)
	2	7 (58,3%)	10 (27,8%)	14 (38,9%)
	3	0 (0,0%)	6 (16,7%)	8 (22,2%)
pN classification	0	12(100,0%)	25 (69,4%)	20 (55,6%)
	1	0 (0,0%)	5 (13,9%)	8 (22,2%)
	2b	0 (0,0%)	6 (16,7%)	8 (22,2%)

**Fig. 4:**
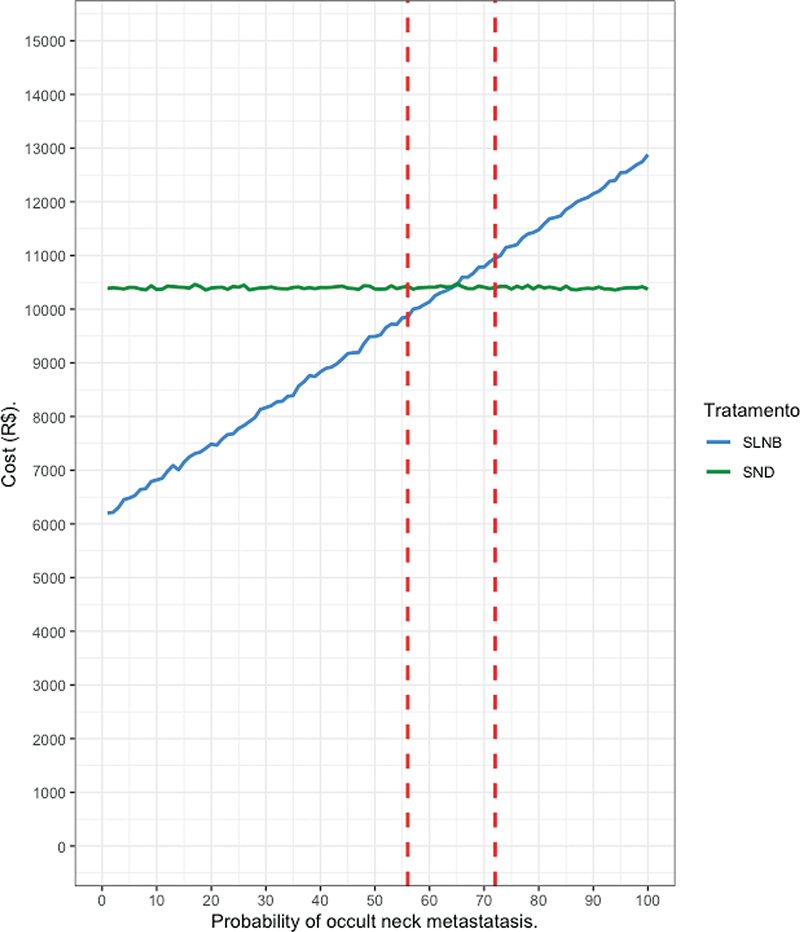
Mean cost of sentinel lymph node biopsy followed by selective neck dissection according to the probability of occult neck metastasis. The vertical lines indicate the interval with no statistically significant difference between the two modalities.

SD: standard deviation; SLNB: sentinel lymph node biopsy; SND: selective neck dissection;

Transition probabilities to different states were defined based on literature data to allow the modeling of a wide range of scenarios. The probability of not finding the sentinel node ranged from 0% 14 to 5.7%. 13 The probability of metastatic SLNB was estimated from public data from the Oncocentro Foundation (Fundação Oncocentro, http://www.fosp.saude.sp.gov.br ). From 8,802 patients with OSCC staged as cT1-2, pathological neck staging was present in 3,427 patients with 316 cases of cervical metastasis (9.2%). Based on this data, we modeled the probability of metastatic SLNB ranging from 9.0% to 100.0%. The probability of not finding the sentinel node varied in 0.5% increments while the probability of metastatic lymph nodes at pathological report in 1.0% increments. For each probability variation, the cost of each strategy was simulated in 1,000 patients and the mean was compared. A progressive increase in the cost of SLNB is observed as more patients are submitted to SND ( Fig. 4 ). In this simulation, the difference in cost of the two strategies becomes non-significant when the transition probability reaches the 56% mark and SLNB becomes more expensive than SND after the 72% mark. It occurs due to the addition of costs from a second in-hospital treatment and increased medical and paramedical postoperative visits and rehabilitation costs.

If this data is applied to the Oncocentro Foundation series, SLNB as initial neck treatment will incur a reduction ranging from 27.93% to 66.54% if we compute the lower cost threshold and upper-cost threshold when compared to SND.

## Discussion

Cancer treatment consumes a significant parcel of healthcare resources due to increasing incidence and raising costs. 19 Therefore, optimization of resource allocation is a major priority and may significantly impact on the number of treated patients.

The impact of SLNB adoption on OSCC treatment costs remains an open question in the literature. An initial analysis in Japan using the Japanese Health Ministerium data shows a significant reduction in costs and patient morbidity with its adoption. In a similar manner to our analysis, the cost of SLNB increases as the incidence of micrometastasis rises. 15 Analyzing data from the European Sentinel Node Trial and using an initial probability of 25% for occult neck metastasis, SLNB caused a significant cost reduction even when all patients were staged as pN + . For cost quantification, data from the British Health System was used. 20 Both simulations share similarities with our model, but significant distinctions may be highlighted. Our model has an outcome comparable to the one found by Kosuda et al, 15 emphasizing the need for rigorous preoperative staging. Gover et al compared five different neck treatment strategies using a public health system payer perspective. In this simulation, SLNB was the most cost-effective strategy. 16 A different model considered fine needle cytologic evaluation of the neck as the first step for neck treatment definition. This simulation considered SLNB as the most cost-effective strategy considering a five-year horizon, but SND when a lifetime horizon was considered. 21 A major difference from other models, including ours, is their use of a Markov model with fixed transition probabilities. Since the risk of neck recurrence is time-dependent, 22 the use of a model not accounting for this information may not be useful in clinical practice.

A significant limitation of this study is the participation of a single center. Therefore, the costs reported reflect the surgical and anesthetic practices of a single institution and do not capture the heterogeneity existing among different practices. This is a finding reported by the SENT trial regarding hospital length of stay. 9 The use of public auction prices means that the calculated price is not that of an institution, but a theoretical construct with greater generality. It also allows to leverage of costs from different institutions by focusing on the items expended and not the price put upon them by health providers.

## Conclusion

The replacement of the SND by SLNB has the potential to significantly decrease the costs associated with the treatment of OSCC patients without sacrificing oncological safety. Considering its incidence and financial impact, more patients could be treated with the same financial and structural resources.
